# Role of soluble urokinase type plasminogen activator receptor (suPAR) in predicting mortality, readmission, length of stay and discharge in emergency patients: A systematic review and meta analysis

**DOI:** 10.1097/MD.0000000000035718

**Published:** 2023-11-10

**Authors:** Syeda Tayyaba Rehan, Hassan ul Hussain, Eman Ali, Kanwal Ashok Kumar, Shehroze Tabassum, Muhammad Hasanain, Asim Shaikh, Gibran Ali, Zohaib Yousaf, Muhammad Sohaib Asghar

**Affiliations:** a Dow University of Health Sciences, Karachi, Pakistan; b King Edward Medical College, Lahore, Pakistan; c Department of Medicine, The Aga Khan University, Karachi, Pakistan; d Division of Pulmonary and Critical Care Medicine, Mayo Clinic - Rochester, NY, USA; e Department of Internal Medicine, Tower Health - Reading Hospital, PA, USA; f Division of Nephrology and Hypertension, Mayo Clinic - Rochester, NY, USA.

**Keywords:** anticoagulant, mortality, predictor, sepsis, Urokinase

## Abstract

**Background:**

Soluble urokinase plasminogen activator receptor (suPAR) is an inflammatory biomarker that is used to predict mortality, readmission, early discharge, and LOS, thus, serves as a useful tool for ED physicians. Our study aims to analyze the efficacy of suPAR in predicting these prognostic markers in ED.

**Methods:**

We performed a comprehensive search on 6 databases from the inception to 30th November 2022, to select the following eligibility criteria; a) observation or triage trial studies investigating the role of suPAR levels in predicting: 30 day and 90-day mortality, 30-day readmission, early discharge (within 24hr), and LOS in patients coming to AMU.

**Results:**

A total of 13 studies were included, with a population size of 35,178, of which 52.9% were female with a mean age of 62.93 years. Increased risk of 30-day mortality (RR = 10.52; 95% CI = 4.82–22.95; I2 = 38%; *P* < .00001), and risk of 90-day mortality (RR = 5.76; 95% CI = 3.35–9.91; I2 = 36%; *P* < .00001) was observed in high suPAR patients. However, a slightly increased risk was observed for 30-day readmission (RR = 1.50; 95% CI = 1.16–1.94; I2 = 54%; *P* = .002). More people were discharged within 24hr in the low suPAR level group compared to high suPAR group (RR = 0.46; 95% CI = 0.40–0.53; I2 = 41%; *P* < .00001). LOS was thrice as long in high suPAR level patients than in patients with low suPAR (WMD = 3.20; 95% CI = 1.84–4.56; I2 = 99%; *P* < .00001).

**Conclusion:**

suPAR is proven to be a significant marker in predicting 30-day and 90-day mortality in ED patients.

## 1. Introduction

According to the Centers for Disease Control and Prevention, about 130 million patients visit the emergency department (ED) in the United States per annum.^[1]^ The basic management of patients in the ED includes triage, registration, treatment, reevaluation, and discharge.^[2]^ It has been documented that 31% of all patients admitted to the ED with initially normal vital signs, show deterioration within the first 24 hours.^[3]^ To execute triage in ED, risk scoring systems,^[4,5]^ as well as traditional triage algorithms based on presenting complaints and vital signs, are used widely.^[4,5]^ Risk stratification models utilizing several biomarkers have proved to be effective in predicting the mortality of incoming ED patients.^[6,7]^ Many prognostic biomarkers including c-reactive protein, procalcitonin, lactate, pro-adrenomedullin, amongst others, have been proposed for clinical decision-making in ED.^[7]^ Worse outcomes in heart failure, acute and chronic kidney disease are found to be associated with elevated suPAR levels.^[8–10]^ suPAR is one of the novel biomarkers, which is released when the cell membrane of immune cells such as activated T-lymphocytes, monocytes, and macrophages are cleaved. Both acute and chronic inflammatory conditions such as sepsis, infectious diseases, malignancies, cardiovascular diseases, organ failures such as kidney and liver failure, and autoimmune diseases show elevated plasma levels of suPAR, which is in contrast to the low levels found in healthy persons.^[11–13]^ Association between suPAR levels and length of hospital stay, shifting of patients to the intensive care unit presenting acutely in ED, and short-term mortality, has been suggested in some studies.^[14–16]^ suPAR levels are considered to be a robust predictor of mortality when combined with the National Early Warning Score, sex, and age of patients in an emergency setting.^[17,18]^

Several observational studies and clinical trials have been conducted to evaluate the role of suPAR in ED outcomes.^[19–23]^ However, there is no pooled result and quantitative synthesis has not been conducted till date. Herein, we conducted a meta-analysis to explore the available data regarding ED outcomes of 30-day mortality, 90-day mortality, readmission within 30 days, discharge within 24 hours, and length of hospital stay (LOS) to draw conclusive results and determine the clinical role of suPAR, as an evolving biomarker, in the prognosis of patients in the emergency setting and its influence on risk stratification.

## 2. Materials and methodology

Guidelines set by the Preferred Reporting Items for Systematic Review and Meta-Analysis were followed to the letter.^[24]^ After a thorough discussion with the members of the research team, we developed a detailed review methodology. This systematic review and meta-analysis has been registered on Prospero (ID: CRD42023406295).

## 3. Data sources and search strategy

A targeted and comprehensive literature search was conducted till November 30th, 2022, using 6 databases, including Pubmed, Google Scholar, Cochrane, Science Direct, Ovid, and ClinicalTrial.gov. Our search was language restricted, and all articles published in languages other than English were excluded. However, our search was kept free from any time restrictions.

Medical Subject Headings (MESH) used were “Emergency Department,” “ER department,” “acute medical unit,” “Soluble Urokinase Plasminogen Activator Receptor levels,” “suPAR levels,” “mortality,” “death,” “readmission,” “rehospitalization,” “discharge,” “Length of stay,” “LOS,” “duration of stay,” “‘hospital stay’” as specified in Supplementary Table S1, http://links.lww.com/MD/K595. Modifications in the search string were made for every database, and a comprehensive search strategy for every single database has been provided in the supplementary file. Manual search for white and gray literature; various data sources, such as editorials, meta-analyses, systematic reviews, conference proceedings for indexed abstracts, and a list of retrieved articles was carried out to spot any relevant studies that may have been missed during our search.

## 4. Study selection and eligibility criteria

The eligibility criteria for inclusion was: experimental group comprising of patients who were admitted to the ED or the acute medical units (AMU) and were clinically monitored using suPAR; the study designs were randomized control trials, retrospective, prospective, single center, registry cohorts, comparative cross-sectional studies, triage trials, pilot observational studies, and post hoc analysis; evaluating the role of suPAR levels in predicting mortality (30, 90 day) readmission (30 days), discharge from hospital within 24 hours and LOS in hospital. Only those observational studies were recruited that set a clear criteria to divide incoming emergency patients into high and low suPAR groups.

After conducting a systematic search, the retrieved articles were exported to the EndNote Reference Library Software (X7 v17.0.0.7072), in order to seek and remove duplicates. Articles were chosen if they met the pre-determined criteria of eligibility. The relevant articles were evaluated by 2 independent reviewers (HUH and STR), initially on the basis of title and abstract and later full text was reviewed in order to confirm the relevance. Group discussion helped in resolving any disparities. Among reviewers, the concordance rate came out to be 97%. All studies recruiting patients other than those admitted through the ED or AMU, case reports, commentaries, review studies, and articles evaluating the prognostic role of suPAR in chronic illness, neurological, and cardiovascular issues, and the role of markers in the prognoses of post-surgical complications were excluded.

## 5. Data extraction and quality assessment

Baseline demographics, study characteristics, and outcome data (30-day mortality, 90-day mortality, readmission within 30 days, discharge within 24 hours, LOS in the hospital were extracted onto a predesigned Microsoft Excel spreadsheet. Two independent reviewers (EA and STR) evaluated the quality of all studies meeting our inclusion criteria. An independent arbiter (HUH) helped in resolving any discrepancies through mutual discussion. Quality assessment studying methodological aspects of observational studies based on the following parameters including selection, comparability, outcome, and exposure, was performed. The risk of bias was assessed for every study included in our meta-analysis. New-castle Ottawa scale (NOS) was used for the quality assessment of included observational studies based on selection, comparability, and outcome/exposure criterion of included studies as shown in Supplementary Table S2, http://links.lww.com/MD/K596.^[25]^ Cross-sectional studies can have a maximum score of 9 and case-control studies can have a maximum score of 10. Two independent reviewers (HUH and STR) independently extracted data and assessed the quality of the included studies and conflicts were resolved by group discussion till consensus.

## 6. Statistical analysis

RevMan (version 5.4; Copenhagen: The Nordic Cochrane Centre, The Cochrane Collaboration, 2014) was used to perform a meta-analysis. Random-effects model was used to pool the results expressed as means ± SD whereas several events occurring in experimental and control groups or inverse variance weighted random-effects model when events were not given, was used to pool the results expressed as risk ratios (RR) with 95% confidence intervals. Forest plots were created to visually assess the pooled results. I² statistics were used to assess heterogeneity (mild heterogeneity = 25%–50%, moderate = 50%–75%, severe heterogeneity > 75%).^[26]^ In all cases, a *P* value < 0.05 was considered statistically significant. Sensitivity analysis was conducted for the assessment of individual study influence on pooled effect size.

## 7. Results

### 7.1. Literature search

**Figure 1.**
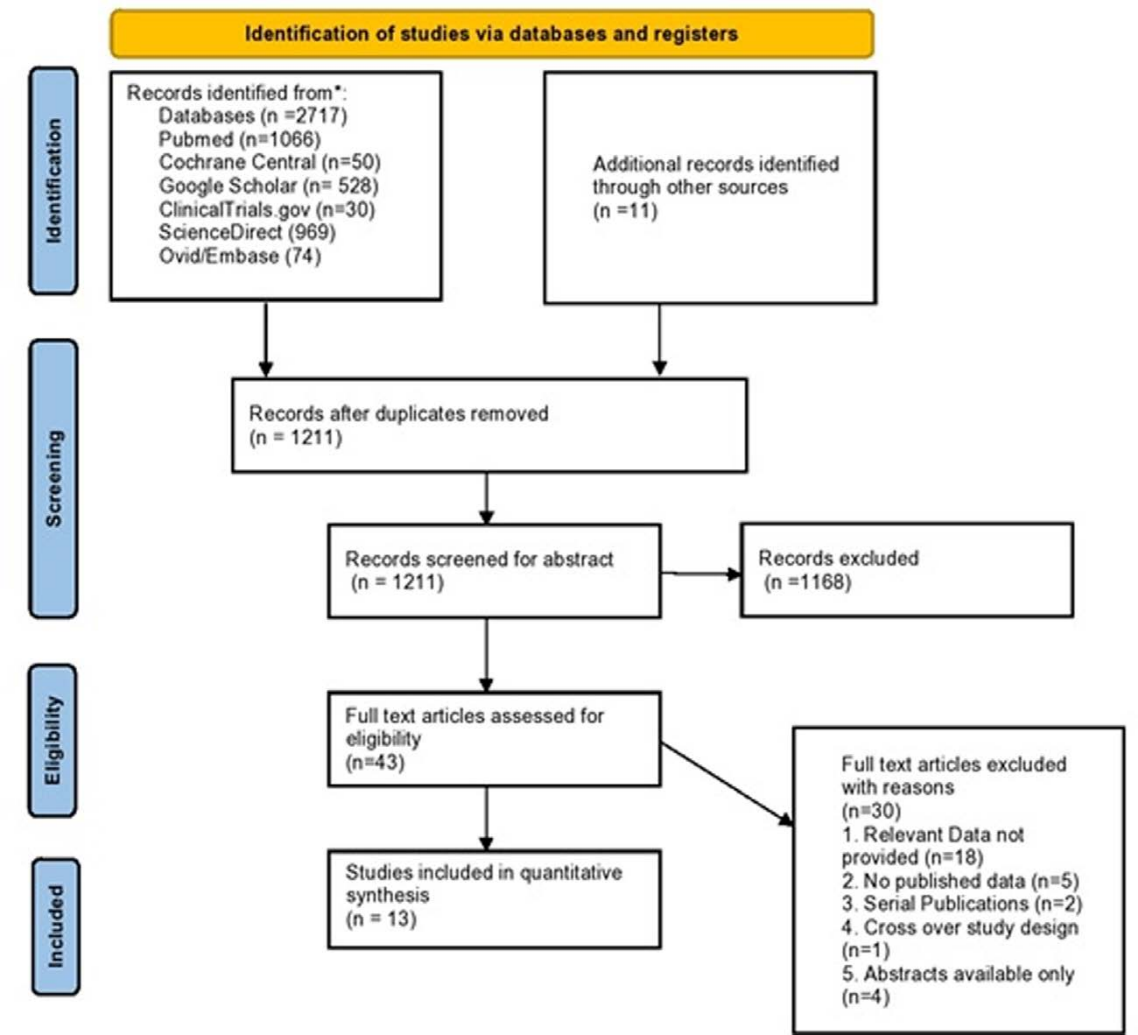
PRISMA flow chart of literature search. PRISMA = Preferred Reporting Items for Systematic Review and Meta-Analysis.

The initial literature search of the 6 databases and other mentioned sources yielded 2717 citations, out of which 1211 citations remained after removal of duplicates. Screening based on title and abstract resulted in the exclusion of 1168 studies. After a full-text assessment of 43 articles for their eligibility, 30 articles were excluded which didn’t meet the inclusion criteria. As a result, 13 studies^[19,21,23,27–36]^ were finalized to be included in this meta-analysis. A summary of the literature search is presented in Figure 1.

### 7.2. Quality assessment

Based on the quality assessment scale, ten cohort observational studies were rated as of “Good” quality. Three studies including Kumar et al 2019, Stauning et al 2021 and Altintas et al 2021 were rated as of “Fair” quality as these studies were awarded zero stars in comparability domain due to lack of comparability of cohorts on the basis of design or analyses controlled for confounders such as age, sex, gender or marital status.^[19,23,29]^ Moreover, Kumar et al 2019 had inadequate duration of follow-up to assess emergency department outcomes in patients under acute care. Summary of quality assessment is presented in Supplementary Table S2, http://links.lww.com/MD/K596.

### 7.3. Study characteristics and patients’ baseline characteristics

Study and patients’ baseline characteristics have been summarized in Tables 1 and 2 respectively. Out of all 13 included studies, 12 are observational studies^[19,21,23,27–33,35,36]^ and 1 is post hoc analysis of a multicenter study.^[34]^ Included studies were published between July 2012 till June 2022. Recruited studies comprised a total of 35,178 participants among which 6004 patients had high suPAR concentration and 18,582 patients who had low suPAR concentration. Out of these 35,178 participants, 18,636 were females and 16,542 were males. The mean age of the participants varied from 45.95 years to 81.75 years, with a mean of 62.93 years. The duration of follow-up varied in every study with the majority of patients being followed up after a duration of 30 or 90 days.

**Table 1:** Characteristics tabulation of included studies.

Author, year	Study type	Region/Hospital	Follow-up duration	suPAR Cutoff (ng/mL)
Uusitalo-Seppala et al 2012	Prospective Cohort	Western Finland/Satakunta Central Hospital	90 d and 1 yr after enrollment = 456.25 d	High = ≥6.4 Low = <6.4
Kumar et al 2019	Pilot Observational non-interventional study	Northern part of India/tertiary care hospital	3 d	High = >5.5, Low = <5.5
Ivic et al 2021	Prospective Obser-vational Cohort	Stockholm Region, Sweden, and Uusimaa Region, Finland./Helsinki University Hospital	-	High = >3.9 Low = <3.9
Stauning et al 2021	Observational Cohort	Copenhagen University Hospital - Hvidovre	14 d	High = >4 Low = <4
Bengaard et al 2021	Registry-based Cohort study	Copenhagen University Hospital Hvidovre, Denmark	90 d	High = >6 Low = <3
Santeri et al 2021	Prospective Observational	Mikkeli Hospital in Finland	30 and 90 d = 120 d	High = >6 Low = <4
Holstein et al 2022^[21]^	Prospective Cohort	Two Finnish hospital regions (Helsinki and Mikkeli)	-	High = >6 Low = <3
Holstein et al 2022^[32]^	Comparative cross sectional	Helsinki University Hospital	7 d	High = >6 Low = <4
Lafon et al 2020	Prospective	International study at 14 EDs	-	-
Chenevier-Gobeaux et al 2021	post hoc analysis from a multicenter study	Three different EDs	30 d	High = ≥3.3 Low = <3.3
Håkansson et al 2019	Retrospective cohort	Copenhagen University Hospital Amager and Hvidovre, Hvidovre, Denmark	30 d	High = >2.6 Low = <2.6
Nayak et al 2015	Prospective observational	Department of Internal Medicine at Bispebjerg & Frederiksberg Hospital, Denmark	3–10 mo = 91.25–304.167 d	High = >4 Low = <4
Altintas et al 2021	Observational cohort	Copenhagen University Hospital, Amager and Hvidovre, Hvidovre, Denmark	14 d	High = ≥6 Low = ≤4

**Table 2:** Population characteristics.

Author, year	Population (N)	High Supar (no of patients)	Low supar (No of patients)	Age (Mean SD)	Female/Male N (%)	SUPAR at admission	Plasma creatinine at admission (Mean SD)	Plasma CRP at admission (Mean SD)
Uusitalo-Seppala et al 2012	539	183	356	60+/−23.67	228 (42.30)/311 (57.69)	>6.4ng/mL, <6.4ng/mL	high supar = 102 (74–148) micromol/L; low supar = 75 (62–92) micromole/L	high supar = 114 (36–212) mg/L; low supar = 110 (36–179) mg/L
Kumar et al 2019	190	126	64	54+/−17.31	110 (57.89)/80 (42.10)	>5.5ng/mL; <5.5ng/mL	-	-
Ivic et al 2021	414	358	56	81.75+/−3.74	234 (56.5%)/180 (43.5)	10.7 (5.8–13.3) ng/mL	-	-
Stauning et al 2021	386	190	196	62.75+/−8.94	221 (57.3)/165 (43)	4.15+/−0.94	77 (62–96.5) micromol/L	-
Bengaard et al 2021	26,291	2538	15,209	56.85+/−9.75	13 845 (52.7) 12446 (47.3)	<3 ng/mL,≥3 to ≤6 ng/mL, >6 ng/mL	-	-
Santeri et al 2021	1747	429	804	69+/−6.34	850 (48.65)/897 (51.4)	4.37+/−0.81 ng/mL	-	-
Holstein et al 2022^[21]^	1858	668	1190	68.75+/−6.62	961 (52%)/897(48.27)	4.37+/−0.81 ng/mL	76 (63–96)	3 (3–19)
Holstein et al 2022^[32]^	330	70	87	66+/−7.50	192 (58.1)/138 (41.81)	4.09+/−0.82 ng/mL	-	4 (4–23) mg/L
Lafon et al 2020	462	124	338	65+/−9.23	210 (45.45)/252 (55)	5.22+/−1.20	-	115 [43–219] mg/L
Chenevier-Gobeaux et al 2021	198	15	183	56.25+/−7.22	69 (34.84)/129 (65)	2.80 (2.17–3.64) ng/mL	86 (71–99) micromol/L	-
Håkansson et al 2019	1341	520	548	45.95+/−9.52	884 (65.92)/457 (34.1)	2.65+/−0.50ng/mL	-	6.0 (2.0–23.0) mg/L
Nayak et al 2015	1036	691	345	69+/−85	611 (59.0)/425 (41.0)	17.35+/−16.65	-	-
Altintas et al 2021	386	92	196	62.75+/−8.94	221 (57.3%)/165 (42.7)	4.15+/−0.94ng/mL	77 (62–96.5) μmol/L	15.5 (2.4–65.8) mg/L

### 7.4. Outcome analysis

All 13 included studies reported the association of high suPAR concentration in patients with different outcomes admitted to the ER. Assessed outcomes included the following:30-day mortality, 90-day mortality, 30-day readmission, discharge within 24 hours, and length of stay within the hospital.

### 7.5. 30-day mortality

**Figure 2.**
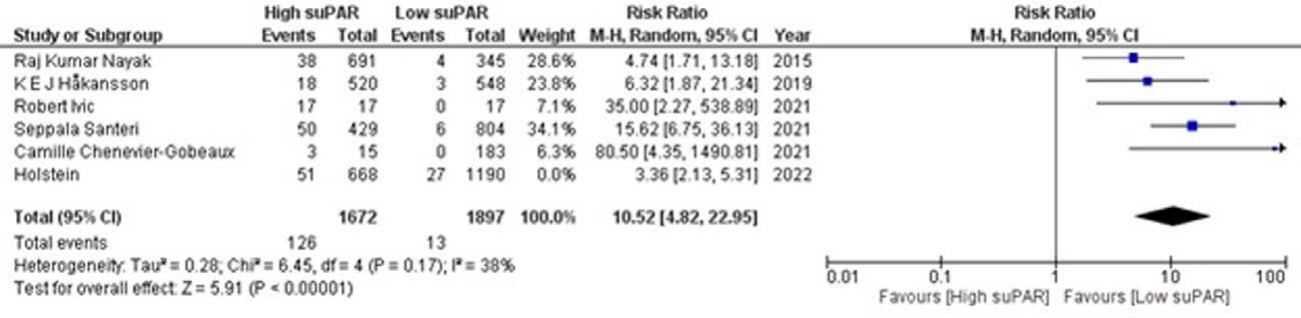
Forest plot for 30 d mortality after sensitivity analysis.

Six studies^[21,28,31,34–36]^ reported data on 30-day mortality in patients having high suPAR levels. There was a positive association between patients with high suPAR concentration and the incidence of 30-day mortality (RR = 8.13; 95% CI = 3.58–18.47; *P* < .00001; I^2^ = 69%) as shown in Supplementary Figure S1, http://links.lww.com/MD/K597. Sensitivity analysis was performed by removing 1 study,^[21]^ which resulted in a significant change in risk (RR = 10.52; 95% CI = 4.82–22.95; *P* < .00001) and revealed mild heterogeneity of the included studies (I^2^ = 38%; *P* = .17) (Fig. 2). This shows that patients having high suPAR levels demonstrate 10.52 times increased risk of having mortality within 30 days as compared to the patients with low suPAR values.

### 7.6. 90-day mortality

**Figure 3.**
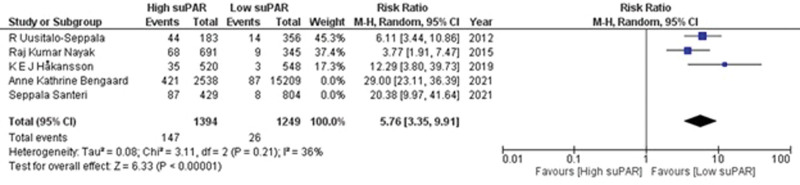
Forest plot for 90 d mortality after sensitivity analysis.

Out of 16 included studies, 5 studies^[27,30,31,35,36]^ provided data on the outcome of 90-day mortality. There were significantly higher odds of risks of 90-day mortality in patients with high suPAR levels as compared to those with low suPAR levels (RR = 11.23; 95% CI = 4.48–28.12; *P* < .00001; I^2^ = 92%) as shown in Supplementary Figure S2, http://links.lww.com/MD/K598. Sensitivity analysis was performed by removing 2 studies,^[30,31]^ this yielded a change in risk (RR = 5.76; 95% CI = 3.35–9.91; *P* < .00001) as well as mild heterogeneity between the studies (I^2^ = 36%; *P* = .21). (Fig. 3) This demonstrates that patients with high suPAR levels have 5.76 times higher chances of the occurrence of mortality within 90 days in comparison with patients with low suPAR concentration.

### 7.6. 30-day readmission

**Figure 4.**
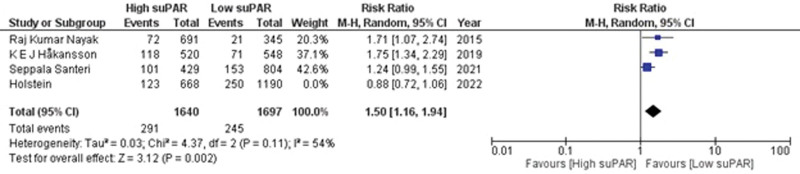
Forest plot for readmission within 30 d after sensitivity analysis.

Adequate data for 30-day readmission was provided in 4 observational studies.^[21,31,35,36]^ The pooled analysis showed an insignificant interdependence between the patients with high suPAR levels and the need for readmission within 30 days (RR = 1.31; 95% CI = 0.93–1.84; *P* = .12; I^2^ = 85%) as shown in Supplementary Figure S3, http://links.lww.com/MD/K599. Exclusion of 1 study^[21]^ by sensitivity analysis revealed a significant risk (RR = 1.50; 95% CI = 1.16–1.94; *P* = .002) and generated mild heterogeneity of the included studies (I^2^ = 54%; *P* = .11) (Fig. 4). It revealed that patients with high suPAR levels have higher chances of getting readmitted to the hospital within 30 days as compared to patients with low suPAR levels.

### 7.7. Discharge within 24 hours

**Figure 5.**
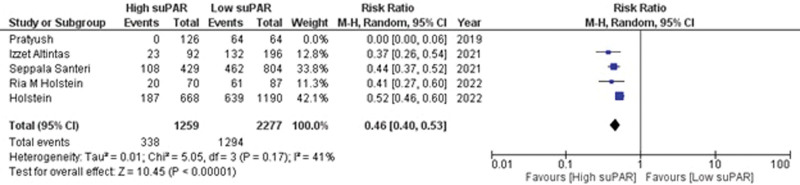
Forest plot for discharge within 24 h after sensitivity analysis.

Data on discharge within 24 hours from the hospital were reported by 5 observational studies.^[19,21,23,31,32]^ This revealed a positive correlation between patients with high suPAR levels and discharge from the hospital within 24 hours (RR = 0.42; 95% CI = 0.32–0.56; *P* < .00001; I^2^ = 80%) as shown in Supplementary Figure S4, http://links.lww.com/MD/K600. Sensitivity analysis was carried out by removing 1 study,^[19]^ this resulted in a very slight alteration in the risk (RR = 0.46; 95% CI = 0.40–0.53; *P* < .00001) and revealed mild heterogeneity (I^2^ = 41%; *P* = .17) (Fig. 5). This demonstrated that patients with high suPAR levels are 0.46 times less likely to discharge within 24 hours from the hospital in comparison to patients with low suPAR levels.

### 7.8. Length of hospital stay

**Figure 6.**
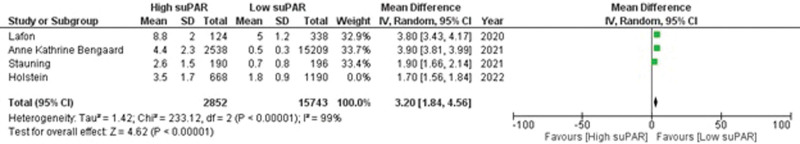
Forest plot for the length of hospital stay after sensitivity analysis.

Four observational studies^[21,29,30,33]^ reported data on the length of hospital stay of patients with high suPAR levels. The pooled analysis revealed a significant association between patients with high suPAR levels and length of hospital stay as compared to patients with low suPAR levels. It showed that patients with high suPAR levels stayed for a greater duration in the hospital as compared to patients with low suPAR levels (WMD = 2.82 days; 95% CI = 1.46–4.19; *P* < .0001; I^2^ = 100%) as shown in Supplementary Figure S5, http://links.lww.com/MD/K601. The exclusion of 1 study^[21]^ was done to perform sensitivity analysis, however, it did not lead to a significant change in the heterogeneity of the included studies (Fig. 6). A consistently high heterogeneity of 99% was observed amongst the studies even after performing sensitivity analysis. The primary reason for this heterogeneity is the massive difference between the population sizes of Bengaard et al^[30]^ and the rest of the studies.^[29,33]^ Additionally, the contrasting methodology concerning the measurement of suPAR levels might have contributed to this extreme heterogeneity.

## 8. Discussion

In this meta-analysis, we discussed the impact of high concentrations of a biomarker known as suPAR on ED outcomes from 13 high-quality observational studies. Our meta-analysis showed that high suPAR concentrations were significantly associated with 30-day mortality, 90-day mortality, 30-day readmission, discharge within 24 hours and length of hospital stay in emergency patients. Despite the frequent research on examining the impact of suPAR concentrations on patients admitted to ED department, no meta-analysis has been conducted to explore this association till date. Thus, we conducted the first ever meta-analysis to examine the impact of suPAR levels as a biomarker in patients admitted under acute care. High suPAR concentrations were associated with increased risk by 10.52 (*P* < .00001), 5.76 (*P* < .00001), 1.50 (*P* = .002), and 2.82 (*P* < .0001) times in 30 and 90-day mortality, 30-day readmission and length of hospital stay, respectively. Our analysis also revealed that high suPAR concentration was associated with lesser rates of discharge from hospital within 24 hours (*P* < .00001).

Acute medical settings have used a variety of biological indicators. It has become evident that biological markers are crucial for the early categorization and prognoses of illnesses. SuPAR is a novel biological marker of immunological activation that is based on endothelial cells and is the soluble form of the urokinase-type plasminogen activator receptor (uPAR). The secondary structure of suPAR is composed of 17 antiparallel sheets connected by 3 short helices. It appears in D1, D2, and D3—three homologous domains.^[11]^ suPAR is a nonspecific biomarker of inflammation and a potent predictor of prognoses for several diseases.^[11]^ Proteases that cleave uPAR from the cell surface convert the receptor into its soluble form (suPAR). The degree of immunological activation is therefore thought to be represented by plasma suPAR levels.^[37]^ Elevated circulating suPAR levels are risk factors for type 2 diabetes, cardiovascular disease, cancer, and overall mortality in the general population, most likely due to low-grade inflammation. Increased plasma suPAR levels have been observed in patients with bacteremia,^[38]^ and high suPAR levels are associated with disease severity and worse prognosis in a variety of diseases, including bacterial meningitis,^[39]^ HIV infection,^[40]^ bacteremia,^[41]^ and active pulmonary tuberculosis.^[42]^ High suPAR levels have been associated with both increased ICU admission and overall lower survival in critically ill patients.^[37]^

Our analysis also revealed that high suPAR levels were significantly associated with increased 30-day readmissions in a population of acute medical patients. Readmissions that are not voluntary, represent a growing issue with complex causes. Readmissions may occur if patients are prematurely discharged, to the wrong location, or if they don’t get sufficient information or support that they need to keep improving. Previous studies have revealed that patients who return to the ED as a result of possible medical error have greater rates of hospital admission than the overall ED population.^[43]^ In contrast, Sabbatini et al and his colleagues reported that patients who experienced an ED visit associated with admission after ED discharge had significantly lower rates of in-hospital mortality, ICU admission, and costs, but higher lengths of stay compared with admissions among patients without a return visit to the ED. These findings imply that inadequacies in the quality of care provided during ED visits may not be accurately reflected by hospital admissions related to ED visits.^[44]^ Unplanned readmissions could also be caused by a breakdown in the coordination of treatment and communication between hospital-based and community-based providers. A readmission indicator could help identify patients who need extra clinical care so that their treatment can be completed before discharge or so that a cross-sectorial effort can be started to stop further readmissions. In addition, there is strong evidence that independent of objective measures of health and other risk factors like socioeconomic status, age, chronic disease, gender, and physical examination, a person assessment of their overall health status is a powerful predictor of morbidity, use of health services, and mortality.^[45]^ Therefore, integration of health assessment tools such as self-rated health (SRH) and self-related worry, with blood serum markers like SUPAR could facilitate identification of individuals with increased risk of hospitalization and mortality.^[46,47]^ As a result, although the association between high suPAR and readmission at the time of admission may not be clinically relevant per se, it does provide evidence that suPAR serves as a measure for disease severity or other pathological conditions.

Numerous biomarkers have been extensively studied for clinical utility, but only a few have proven their significance in clinical settings. Available biomarkers in the clinical realm such as C-reactive protein, Proadrenomedullin (MR-proADM), serum lactate, and procalcitonin have significantly influenced clinical decision making and changed the ways in which we practice medicine.^[48]^ Many studies have investigated these biomarkers and they have proven to be successful in predicting ED outcomes.^[49–53]^ In addition, a few studies have compared the prognostic ability of suPAR levels with other available biomarkers. A blinded prospective cohort conducted to compare the prognostic ability of suPAR and serum lactate biomarkers showed that high suPAR above 9ng/mL had a significant predictive ability of 30-day mortality (*P* < .001) whereas lactate was not a significant predictor of 30-day mortality (*P* = .269).^[28]^ Moreover, another observational study which compared suPAR alone or in combination with other prognostic biomarkers including C-reactive protein/lactate could not predict ED discharge or hospital admission among 109 patients.^[54]^ The findings have been contradictory in studies published until now. Thus, additional research is also needed to compare the predictive accuracy of suPAR concentrations with other approved biomarkers and improve clinical decision making.

Our paper not only confirms but also contributes with new knowledge of suPAR as an independent predictor of length of hospital stay and discharge within 24 hours. In our analysis, we observed a 2.82 times increase in the length of hospital stay among high suPAR patients under acute care. Therefore, predicted patient prognosis derived from observations of suPAR concentration may influence clinical decision making, and perhaps possibly impact outcomes. However, it should be emphasized that currently there is no data to support that knowledge of the prognosis of a particular patient can lead to a favorable change in outcome. Additional future research now should also be directed on how suPAR can be utilized in improving patient outcomes. Perhaps future studies can look into specific system disease based stratification of suPAR outcomes so management strategies can be tailored to the individual patient.

## 9. Strengths and limitations

Our study had some considerable strengths. Firstly, we systematically reviewed literature published till date which evaluated ED outcomes in high suPAR patients under acute care. Secondly, to our knowledge, this is the first study which comprehensively includes all published randomized control trials and observational studies of high suPAR patients and low suPAR patients under acute care. However, some limitations of our study include the inability to investigate more sub-groups as we used study-level data instead of patient-level data. In addition, it has not been determined whether the use of a prognostic biomarker for risk stratification in the ED translates into meaningful and prognosis-changing interventions. A risk-scoring system useful in emergency medicine must be simple, easily obtainable, and quickly translatable to clinical decision-making. Lastly, the included observational studies have their own criteria to divide the emergency influx into high and low suPAR groups instead of following any universal criteria. Thus studies, even though having closely followed scaling to categorize high and low suPAR groups, show differences in the high and low suPAR levels which makes it difficult to conclude the cutoff.

### 9.1. Conclusion

High suPAR level at admission to ED is a sign of a serious condition and is attributed to higher risks of mortality and readmission. Plasma suPAR levels may thus be useful for evaluating medical patients who have been admitted to the AMU to ascertain whether they need a more thorough clinical evaluation as well as intensive monitoring and care. Further trials and investigations are needed to clinically approve the prognostic use of suPAR in emergency settings.

## Author contributions

**Conceptualization:** Syeda Tayyaba Rehan.

**Data curation:** Hassan ul Hussain.

**Formal analysis:** Eman Ali.

**Investigation:** Kanwal Ashok Kumar.

**Methodology:** Shehroze Tabassum.

**Project administration:** Zohaib Yousaf, Muhammad Sohaib Asghar.

**Resources:** Muhammad Hasanain.

**Software:** Asim Shaikh.

**Supervision:** Gibran Ali.

**Validation:** Gibran Ali.

**Visualization:** Zohaib Yousaf.

**Writing – original draft:** Asim Shaikh.

**Writing – review & editing:** Muhammad Sohaib Asghar.

## Supplementary Material














