# Supplementary material for: Role of soluble urokinase type plasminogen activator receptor (suPAR) in predicting mortality, readmission, length of stay and discharge in emergency patients: A systematic review and meta analysis
Source: Medicine (Baltimore). 2023 Nov 10;102(45):e35718. doi: 10.1097/MD.0000000000035718 (PMC10637562; doi:10.1097/MD.0000000000035718)
Supplement: Supplementary file 1 [file medi-102-e35718-s001.docx]

**Supplementary Table 1: Search strategy used in each database searched.**

| **Database** | **Search Strategy** | **Articles retrieved** |
| --- | --- | --- |
| Pubmed | ((("solubility"[MeSH Terms] OR "solubility"[All Fields] OR "solubilities"[All Fields] OR "soluble"[All Fields] OR "solubles"[All Fields] OR "solublization"[All Fields] OR "solublize"[All Fields] OR "solublized"[All Fields]) AND ("receptors, urokinase plasminogen activator"[MeSH Terms] OR ("receptors"[All Fields] AND "urokinase"[All Fields] AND "plasminogen"[All Fields] AND "activator"[All Fields]) OR "urokinase plasminogen activator receptors"[All Fields] OR ("urokinase"[All Fields] AND "plasminogen"[All Fields] AND "activator"[All Fields] AND "receptor"[All Fields]) OR "urokinase plasminogen activator receptor"[All Fields])) OR "suPAR"[All Fields] OR (("emergency service, hospital"[MeSH Terms] OR ("emergency"[All Fields] AND "service"[All Fields] AND "hospital"[All Fields]) OR "hospital emergency service"[All Fields] OR ("emergency"[All Fields] AND "department"[All Fields]) OR "emergency department"[All Fields]) AND ("serum"[MeSH Terms] OR "serum"[All Fields] OR "serums"[All Fields] OR "serum s"[All Fields] OR "serumal"[All Fields]) AND ("biomarker s"[All Fields] OR "biomarkers"[MeSH Terms] OR "biomarkers"[All Fields] OR "biomarker"[All Fields]))) AND ("mortality"[MeSH Terms] OR "mortality"[All Fields] OR "mortalities"[All Fields] OR "mortality"[MeSH Subheading] OR ("death"[MeSH Terms] OR "death"[All Fields] OR "deaths"[All Fields]) OR ("readmission"[All Fields] OR "readmissions"[All Fields]) OR ("discharges"[All Fields] OR "discharging"[All Fields] OR "patient discharge"[MeSH Terms] OR ("patient"[All Fields] AND "discharge"[All Fields]) OR "patient discharge"[All Fields] OR "discharge"[All Fields] OR "discharged"[All Fields]) OR ("length of stay"[MeSH Terms] OR ("length"[All Fields] AND "stay"[All Fields]) OR "length of stay"[All Fields])) AND ("emergency service, hospital"[MeSH Terms] OR ("emergency"[All Fields] AND "service"[All Fields] AND "hospital"[All Fields]) OR "hospital emergency service"[All Fields] OR ("emergency"[All Fields] AND "department"[All Fields]) OR "emergency department"[All Fields] OR (("acute"[All Fields] OR "acutely"[All Fields] OR "acutes"[All Fields]) AND ("assess"[All Fields] OR "assessed"[All Fields] OR "assessement"[All Fields] OR "assesses"[All Fields] OR "assessing"[All Fields] OR "assessment"[All Fields] OR "assessment s"[All Fields] OR "assessments"[All Fields]) AND "unit"[All Fields]) OR (("acute"[All Fields] OR "acutely"[All Fields] OR "acutes"[All Fields]) AND ("medic"[All Fields] OR "medical"[All Fields] OR "medicalization"[MeSH Terms] OR "medicalization"[All Fields] OR "medicalizations"[All Fields] OR "medicalize"[All Fields] OR "medicalized"[All Fields] OR "medicalizes"[All Fields] OR "medicalizing"[All Fields] OR "medically"[All Fields] OR "medicals"[All Fields] OR "medicated"[All Fields] OR "medication s"[All Fields] OR "medics"[All Fields] OR "pharmaceutical preparations"[MeSH Terms] OR ("pharmaceutical"[All Fields] AND "preparations"[All Fields]) OR "pharmaceutical preparations"[All Fields] OR "medication"[All Fields] OR "medications"[All Fields]) AND "unit"[All Fields]) OR ("acute care"[Journal] OR ("acute"[All Fields] AND "care"[All Fields]) OR "acute care"[All Fields])) | 1066 |
| Cochrane Library | (Soluble Urokinase Plasminogen Activator Receptor OR suPAR OR emergency department serum biomarker) AND (mortality OR death OR readmission OR discharge OR length of stay) AND (Emergency department OR acute assessment unit OR acute medical unit OR acute care) | 50 |
| Clinicaltrials.gov | (Soluble Urokinase Plasminogen Activator Receptor) | 30 |
| Google Scholar | (Soluble Urokinase Plasminogen Activator Receptor OR suPAR OR emergency department serum biomarker) AND (mortality OR death OR readmission OR discharge OR length of stay) AND (Emergency department OR acute assessment unit OR acute medical unit OR acute care) | 528 |
| ScienceDirect | (Soluble Urokinase Plasminogen Activator Receptor OR suPAR ) AND (mortality OR death OR readmission OR discharge OR length of stay) AND (Emergency department OR acute assessment unit) | 969 |
| Ovid | (Soluble Urokinase Plasminogen Activator Receptor OR suPAR OR emergency department serum biomarker) AND (mortality OR death OR readmission OR discharge OR length of stay) AND (Emergency department OR acute assessment unit OR acute medical unit OR acute care) | 74 |
|  |  |  |
