# Supplementary material for: Role of soluble urokinase type plasminogen activator receptor (suPAR) in predicting mortality, readmission, length of stay and discharge in emergency patients: A systematic review and meta analysis
Source: Medicine (Baltimore). 2023 Nov 10;102(45):e35718. doi: 10.1097/MD.0000000000035718 (PMC10637562; doi:10.1097/MD.0000000000035718)
Supplement: Supplementary file 2 [file medi-102-e35718-s002.docx]

**Supplementary Table 2: Quality Assessment of Included Studies via Newcastle Ottawa Scale**

| STUDY ID | SELECTION ^a^ | | | | COMPARABILITY ^b^ | OUTCOME ^c^ | | | QUALITY ASSESSMENT BASED ON AHRQ ^d^ |
| --- | --- | --- | --- | --- | --- | --- | --- | --- | --- |
|  | Representativeness of the exposed cohort | Selection of non-exposed cohort | Ascertainment of exposure | Demonstration that outcome of interest was not present at the start of the study | Comparability of cohorts on basis of design or analysis controlled for confounders | Assessment of outcome | Adequacy of the duration of follow up | Adequacy of completeness of follow up |  |
| Pratyush et al. 2019 | * | * | * | * | - | * | - | * | Fair |
| Ivic et al. 2021 | * | * | * | * | ** | * | * | * | Good |
| Holstein et al. 2022 (1) | * | * | * | * | * | * | * | * | Good |
| Seppala et al. 2012 | * | * | * | * | ** | * | * | * | Good |
| Holstein et al. 2022 (2) | * | * | * | * | ** | * | * | * | Good |
| Stauning et al. 2021 | * | * | * | * | - | * | * | * | Fair |
| Bengaard et al. 2022 | * | * | * | * | ** | * | * | * | Good |
| Santeri et al. 2021 | * | * | * | * | ** | * | * | * | Good |
| Lafon et al. 2020 | * | N/A | * | * | ** | * | * | * | Good |
| [Chenevier-Gobeaux](https://pubmed.ncbi.nlm.nih.gov/?term=Chenevier-Gobeaux+C&cauthor_id=33662350) et al. 2021 | * | * | * | * | * | * | * | - | Good |
| Hakansson et al. 2019 | * | * | * | * | ** | - | * | * | Good |
| Nayak et al. 2015 | * | * | * | * | ** | * | * | * | Good |
| Altintas et al. 2021 | * | * | * | * | - | * | * | * | Fair |

^a,b,c^ The higher the number of asterisk (*), the better quality of a given criterion. The maximum asterisks possible for each domain are selection = 3, comparability =2, outcome = 3. ^d^The Newcastle Ottawa Scale for Cohort studies converted to AHRQ standards (good, fair, poor). AHRQ: Agency for Healthcare Research and Quality.
