# Supplementary figures and images for: Role of soluble urokinase type plasminogen activator receptor (suPAR) in predicting mortality, readmission, length of stay and discharge in emergency patients: A systematic review and meta analysis
Source: Medicine (Baltimore). 2023 Nov 10;102(45):e35718. doi: 10.1097/MD.0000000000035718 (PMC10637562; doi:10.1097/MD.0000000000035718)

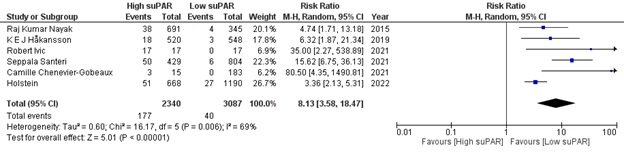

Supplement: Supplementary file 3 [file medi-102-e35718-s003.tiff]

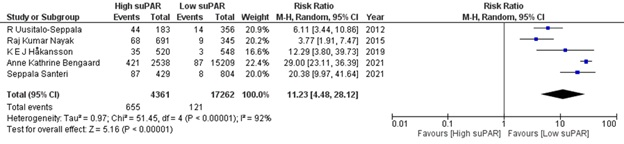

Supplement: Supplementary file 4 [file medi-102-e35718-s004.tiff]

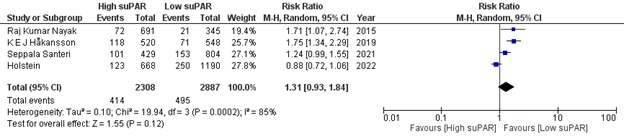

Supplement: Supplementary file 5 [file medi-102-e35718-s005.tiff]

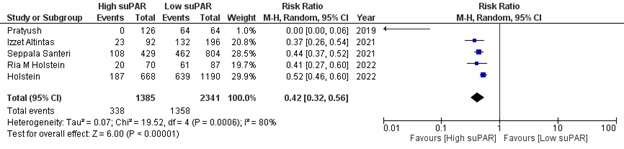

Supplement: Supplementary file 6 [file medi-102-e35718-s006.tiff]

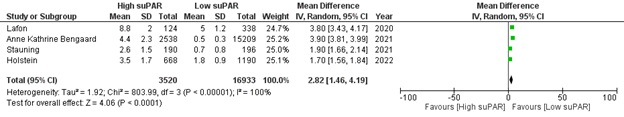

Supplement: Supplementary file 7 [file medi-102-e35718-s007.tiff]
